# Constructing covalent organic frameworks in water *via* dynamic covalent bonding

**DOI:** 10.1107/S2052252516013762

**Published:** 2016-09-14

**Authors:** Jayshri Thote, Harshitha Barike Aiyappa, Raya Rahul Kumar, Sharath Kandambeth, Bishnu P. Biswal, Digambar Balaji Shinde, Neha Chaki Roy, Rahul Banerjee

**Affiliations:** a Physical/Materials Chemistry Division, CSIR-National Chemical Laboratory, Dr Homi Bhabha Road, Pune, Maharashtra 411008, India; b Academy of Scientific and Innovative Research, New Delhi 110020, India; c Indian Institute of Science Education and Research, Pune, Maharashtra 411008, India; d Centre for Research in Nanotechnology and Science, Indian Institute of Technology Bombay, Mumbai 400076, India

**Keywords:** crystalline porous polymers, hydrothermal, porous organic solids, hydrogen bonding, microporous materials

## Abstract

The formation of keto-enamine based crystalline, porous polymers in water is investigated for the first time. Facile access to the Schiff base reaction in water has been exploited to synthesize stable porous structures using the principles of Dynamic Covalent Chemistry (DCC). Most credibly, the water-based Covalent Organic Frameworks (COFs) possess chemical as well as physical properties such as crystallinity, surface area and porosity, which is comparable to their solvothermal counterparts. The formation of COFs in water is further investigated by understanding the nature of the monomers formed using hydroxy and non-hydroxy analogues of the aldehyde. This synthetic route paves a new way to synthesize COFs using a viable, greener route by utilization of the DCC principles in conjunction with the keto–enol tautomerism to synthesize useful, stable and porous COFs in water.

## Abstract 2

The present work effectively highlights the utilization of Dynamic Covalent Chemistry (DCC) principles in conjunction with the keto–enol tautomerism to synthesize useful, stable, crystalline and porous Covalent Organic Frameworks (COFs) in water, which thereby merits over the conventional solvothermal COF synthesis protocol with its simpler and greener appeal.

## 1. Introduction   

Crystalline porous polymers, also known as Covalent Organic Frameworks (COFs) and Covalent Triazine Frameworks (CTFs), have gained significant scientific attention in recent years due to their utility in gas storage, catalysis, proton conduction and sensing (Dalapati *et al.*, 2013 ▸; Uribe-Romo *et al.*, 2011 ▸; Wu *et al.*, 2012 ▸; Bhunia *et al.*, 2013 ▸; Chang *et al.*, 2013 ▸; Song *et al.*, 2013 ▸). However, poor hydrolytic stability and the constraints involved in COF synthesis limit their exploration towards any practical application. Notably, the idea behind the COF synthesis is inspired by the dynamic covalent chemistry principles which involve the use of reversible condensation reactions such as boronic acid trimerization, boronate ester formation and Schiff base reactions (Zwaneveld *et al.*, 2008 ▸; Côté *et al.*, 2007 ▸; Belowich & Stoddart, 2012 ▸; DeBlase *et al.*, 2013 ▸). The dynamic features and reversibility of such reactions allow the structural units to self-construct, thereby instigating long-range periodicity and crystallinity in COFs (Bojdys *et al.*, 2010 ▸; Bildirir *et al.*, 2015 ▸). However, it is equally important to note that the same reversibility also facilitates the undesired back reaction and as a result, the boronic acid, boronate ester and Schiff base derived COFs decompose readily in water. It has been observed that the introduction of proton tautomerism induces high chemical stability (Kandambeth *et al.*, 2012 ▸) during the course of COF formation. Even then, the scalable synthesis of COFs is still at infancy as the synthetic processes demand vigilant set-up conditions such as (*a*) a Pyrex tube sealed under high vacuum, (*b*) an inert atmosphere, (*c*) freeze–pump–thaw cycles and most importantly, (*d*) the use of high boiling organic solvents such as *N*,*N*-dimethylformamide (DMF), *N*,*N*-dimethylacetamide (DMAc), *o*-dichlorobenzene (DCB), mesitylene, 1,4-dioxane *etc.* (Côté *et al.*, 2005 ▸). In addition, it also demands a high temperature (120–150°C) and long reaction time (48–72 h). The sparing solubility of individual reagents in a particular solvent system and their slow diffusion into another solvent system precisely controls the crystallization. Furthermore, these features also facilitate the nucleation of the crystalline materials within a closed reaction vessel wherein the presence of water plays a vital role in maintaining the reversibility of the reaction for further crystal growth (Liu *et al.*, 2013 ▸). These synthetic hurdles limit the COF synthesis to milligram scale apart from the extensive use of toxic organic solvents making the synthetic procedure intricate and environmentally malignant. Even though there are few reports on the synthesis of COFs using the scalable solvent-free mechanochemical route, the poor surface area and the low crystallinity of the as-synthesized materials makes them unworthy of any potential applications (Biswal *et al.*, 2013 ▸; Shinde *et al.*, 2016 ▸). Water being a universal solvent is undoubtedly the most obvious choice for any ‘clean’ reaction. The synthesis of Schiff-based COFs in water would thus be an interesting addition to the dynamic covalent reaction toolbox. Although scalable synthesis of a few porous materials, namely metal–organic frameworks (MOFs), zeolites, porous silica, are reported in water (Meng & Xiao, 2014 ▸; Sánchez-Sánchez *et al.*, 2015 ▸), the concept of water-based COF synthesis is unprecedented as water being the end product could essentially drive back the dynamic reversible imine (—C=N—) bond formation in water (Dai *et al.*, 2016 ▸; Zhoua *et al.*, 2016 ▸). Dynamic Covalent Chemistry has been extensively employed for COF synthesis which involves the self-correction mechanism to form crystalline COFs. However, to the best of our knowledge, the use of dynamic imine chemistry for the synthesis of COFs ‘in water’ has not yet been demonstrated. Herein we have synthesized a series of COFs: TpPa-1, TpPa-2, TpBD, TpBpy and DAAQ in water. In order to further validate our protocol, we have also synthesized a new COF, TpFn, in water as well. The as-synthesized keto-enamine based COFs were found to be crystalline, porous and chemically stable as similar to their solvothermal counterparts that were conventionally synthesized *via* a vacuum-sealed tube technique.

## 2. Experimental   

**Figure 1:**
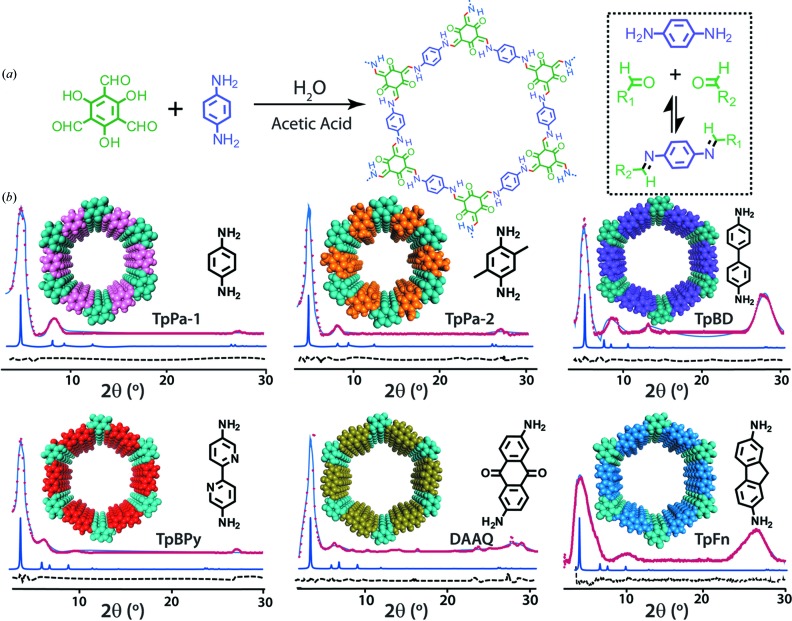
(*a*) Schematic representation of the synthesis of porous crystalline polymers in water; (*b*) comparative simulated (violet), refined (blue), experimental (red) and difference (black dashed curve) PXRD patterns of the COFs synthesized in water.

The syntheses of TpPa-1, TpPa-2, TpBD, TpFn, DAAQ and TpBpy were performed *via* Schiff-base condensation of 1,3,5-triformylphloroglucinol (Tp), and corresponding amines, namely *p*-phenylenediamine (Pa-1)/2,5-dimethyl-*p*-phenylene­diamine (Pa-2)/biphenyl-4,4′-diamine (BD)/2,7-diaminofluorene (Fn)/2,6-diaminoanthraquinone (AQ)/2,2′-bipyridine-4,4′-diamine (Bpy) in water and acetic acid medium (Fig. 1 ▸). The mixture was heated at 120°C for 3 d and finally washed with copious amounts of water and ethanol.

## 3. Results and discussion   

The PXRD patterns of the as-synthesized COFs (TpPa-1 and -2) showed an intense first peak at 2θ 4.7, whereas for TpBD, TpBpy, DAAQ and TpFn it appeared at 2θ 3.4, both corresponding to the 100 plane reflection (Fig. 1 ▸ *b*), signifying their high degree of crystallinity similar to their conventional solvothermal and mechnochemically synthesized counterparts (Figs. S2 and S3). The π–π stacking distance between the successive COF layers was found to range between 3.8 and 4.4 Å, as calculated using the *d* spacing between 001 planes. The FT–IR spectra indicated the disappearance of the N—H stretching frequency (3100–3300 cm^−1^, corresponding to the free diamines) and the carbonyl stretching frequency (1639 cm^−1^, corresponding to the aldehyde carbonyl) in all the COFs synthesized in water. In addition, the appearance of peaks corresponding to the stretching of the —C=C— and —C—N— bonds confirmed the successful formation of COFs in water (Fig. S4). The enol–imine tautomerism was further confirmed using ^13^C CP-MAS solid-state NMR spectra. In accordance with their seal-tube synthesized counterparts, the ^13^C NMR of the trialdehyde showed a prominent carbonyl peak at δ 192 p.p.m. (corresponding to the —CHO aldehydic peak), while that of the resulting COF indicated the presence of the carbonyl peak at δ 172–188 p.p.m. (corresponding to the —C=O keto group). The absence of the signal corresponding to aldehydic carbon thereby certifies the completion of the reactions in water, thus signifying the ability of water to potentially bring about such reactions (Fig. S5).

**Figure 2:**
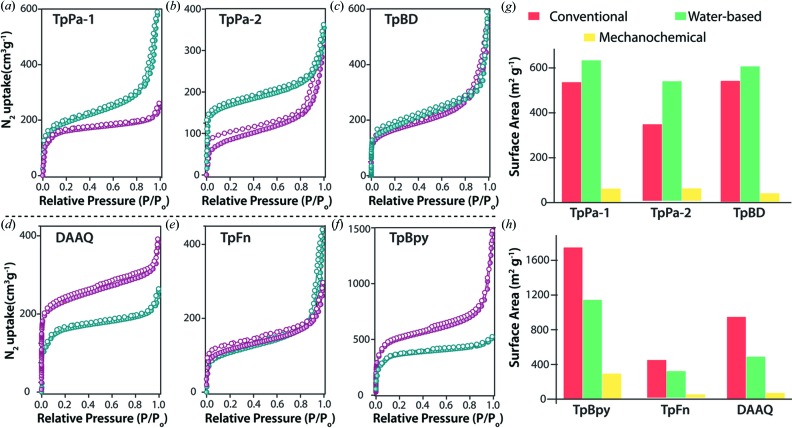
(*a*)–(*f*) N_2_ adsorption isotherms of COFs synthesized using the conventional (purple) and water-based (blue) route; (*g*) and (*h*) comparative chart indicating the surface area of the COFs synthesized in water.

**Figure 3:**
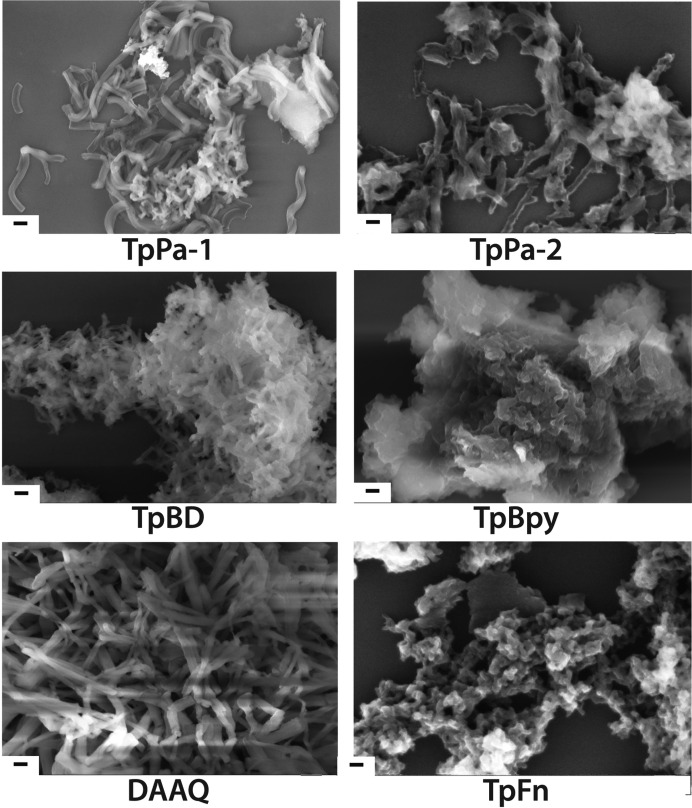
SEM images of the COFs synthesized in water (scale bar = 200 nm).

The chemical stability of each of the COFs was checked in 9 N HCl and 3 N NaOH for 3 d. It was observed that all the PXRD peak positions were retained and no extraneous peaks were observed, indicating the retention of the framework structure after the chemical treatment (Figs. S7 and S9). However, the increase in the 001 plane peak intensity in a few of the COFs (corresponding to the π–π stacking in COFs) hints at the possible exfoliation of the COF sheets during the chemical treatment. Accordingly, the FT–IR spectra also indicated the retention of the —C=C and —C—N stretching peaks after the chemical treatment (Figs. S8 and S10). The TGA profile of TpFn indicated thermal stability up to ∼ 300°C, after which a gradual weight loss up to ∼ 60% was observed (Fig. S11). Furthermore, the porosity and the surface area of activated COFs was evaluated using the Brunauer–Emmett–Teller (BET) model. The newly introduced TpFn COF showed a Type II N_2_ adsorption isotherm with a surface area of 302 m^2^ g^−1^. The surface area of TpPa-1, TpPa-2, TpBD, TpFn, TpBpy, DAAQ was found to be 633, 530, 601, 354, 1140 and 489 m^2^ g^−1^, respectively (Figs. 2 ▸ *a*–*f*). In the study, it can be explicitly observed that the surface areas of the COFs constructed in water are relatively lower compared with their solvothermal counterparts. This is believed to be a direct consequence of the effect of reactant solubility on the framework formation. This effect is very much evident in cases of TpBpy and TpBD, wherein the 2, 2′ positions of the biphenyl ring (in TpBD) are replaced by N atoms (in Bpy). The presence of such nitrogen atoms in the ligand backbone is very much known to favour hydrogen-bonding interactions with acids (Shinde *et al.*, 2016 ▸). This effect is similarly believed to improve the solubility of 2,2′-bipyridine-4,4′-diamine in the acidified water medium, which would eventually enhance the rate of the imine bond formation. However, it is also important to note that although the presence of acetic acid assists the solubilization of the amines, the aldehydic reactant is still largely insoluble in the medium, unlike in the case of solvothermal conditions. Thus, the poor solubility of the reactants could be seen as a major factor that imposes less surface area to the forming structures. However, convincingly, the surface area of the as-synthesized COFs is still significantly higher than their other widely accepted also greener, mechanochemically synthesized counterparts. The high degree of crystallinity and surface area of the resulting COFs is indicative of the fact that the π–π stacking is well favoured in aqueous medium. The rest of the COFs (TpFn, TpBpy and DAAQ) showed a marginal decrease in their surface area (Fig. 2 ▸ *h*). The poor solubility of the corresponding amines, 2,7-diaminofluorene (Fn) and 2,6-diaminoanthraquinone (AQ) in aqueous medium is believed to decrease the crystallinity and henceforth the surface area in the case of TpFn and DAAQ COFs. The pore size distribution of COFs indicated the presence of narrow sized pores ranging between 1.4 and 2.0 nm (Fig. S13). The TEM and the SEM analysis revealed the presence of well isolated sheet-like structures in the case of TpPa-1 and TpPa-2 with no observable agglomeration. DAAQ, TpBD and TpBpy had pronounced fibrillar morphology, similar to their solvothermal-synthesized counterparts. The newly synthesized TpFn revealed the formation of petal-shaped structures with an average length of 600 nm and width 220 nm (Figs. 3 ▸ and S12). Among all, TpBpy showed a maximum H_2_ uptake of 108 cm^3^ g^−1^ at 77 K and 1 bar pressure (Fig. S14). The hydrothermally synthesized COFs showed considerable ability to uptake CO_2_ at 273 K: TpBpy (73 cm^3^ g^−1^), DAAQ (82 cm^3^ g^−1^) and TpBD (95 cm^3^ g^−1^) (Fig. S15).

**Figure 4:**
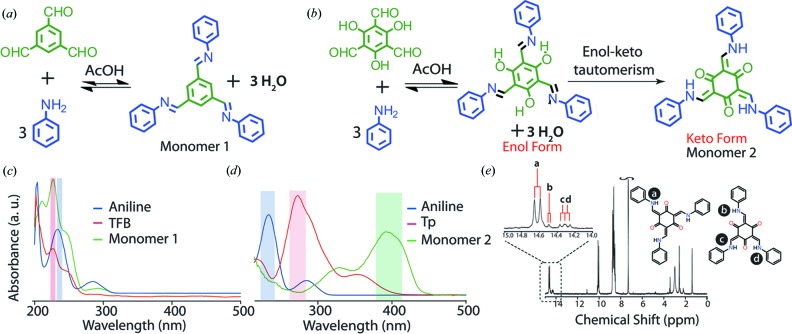
(*a*) and (*b*) Synthetic scheme for the formation of monomer 1 and monomer 2 in water–acetic acid medium; (*c*) and (*d*) comparative UV–vis spectra of the respective monomers with their reactants; (*e*) ^1^H spectrum of monomer 2.

It is important to note that the concept of aqueous synthesis cannot be generalized to COFs derived *via* every dynamic covalent reaction. In particular, this concept cannot be extended to those having B—O, C=N bonds as they are susceptible to reversible back reaction upon exposure to moisture, thereby resulting in their structural collapse. It is known that an important factor rendering chemical stability to the Schiff based COFs is the introduction of —OH functionalities adjacent to the Schiff base centres as previously described. Upon closer analysis, it has been found that in the case of such tautomerized Schiff base reactions the presence of water (which also happens to be a by-product of condensation) does not hamper the proceedings of the reaction as the product formed is water stable as well as water insoluble. In order to investigate the mechanism of the imine and keto-enamine bonded COF formation in water, the time-dependent UV–vis spectroscopic study of the monomer formation was carried out using benzene-1,3,5-tricarbaldehyde and its hydroxyl analogue 2,4,6-trihydroxybenzene-1,3,5-tricarbaldehyde (Figs. 4 ▸ *a* and *b*). Both the precipitates were collected and washed thoroughly using ethanol. The UV–vis spectra of the benzene-1,3,5-tricarbaldehyde and aniline reaction mixture did not show the appearance of any product peaks even after 10 h of reaction time (Fig. 4 ▸ *c*). This indicates the sluggishness of the Schiff base condensation reaction towards the formation of (*N*,*N*′,*N*′′,*E*,*N*,*N*′,*N*′,*E*,*N*,*N*′,*N*′′,*E*)-*N*,*N*′,*N*′′-(benzene-1,3,5 triyltris(methanylylidene))trianiline (monomer 1) in water. On the other hand, its hydroxyl analogue *i.e.* 2,4,6-trihydroxybenzene-1,3,5-tricarbaldehyde reacted readily with aniline to form (2*E*,4*E*,6*E*)-2,4,6-tris­((phenylamino)methylene)cyclohexane-1,3,5-trione (monomer 2) in water. The UV–vis study showed the disappearance of the peaks corresponding to the starting materials (265 nm for 2,4,6-trihydroxybenzene-1,3,5-tricarbaldehyde and 233 nm for aniline) with the appearance alongside of a new peak at 396 nm, indicating the formation of monomer 2 (Chong *et al.*, 2003 ▸). From the ^1^H NMR spectral study of monomer 2 in 1,1,2,2-tetrachloroethane-*d*_2_, the presence of two isomeric forms (*Cs* and *C*3) of keto-enamine could also be observed (Fig. 4 ▸ *e*). In addition, the spectrum also showed the coupling between the enamine proton and —NH_2_. The sluggishness in the rate of monomer 1 formation is possibly due to the reversible nature of the imine bond, which makes it extremely sensitive to water. The presence of a large volume of solvent water molecules in the system is believed to severely retard monomer 1 formation (Sauer *et al.*, 2006 ▸). On the other hand, the Schiff base reaction involving 2,4,6-trihydroxybenzene-1,3,5-tricarbaldehyde (hydroxy aldehyde) is observed to follow a two-step process: a reversible Schiff base reaction forming a Schiff base compound in the first step followed by an irreversible enol to keto tautomerization process yielding a stable keto-enamine compound in the second step (Fig. 4 ▸ *b*). It is worth noting that as the next in line the tautomerization process consumes the Schiff base intermediates formed during the first step of the reversible reaction; the forward reaction is accelerated in accordance with Le Chatelier’s principle. This thereby drives the system towards the second irreversible step involving keto tautomerism. In the case of a hydroxy-containing aldehyde, the insolubility of the resulting monomer 2 is believed to instigate the progress of the reaction in water.

The PXRD spectra were recorded at regular intervals to monitor the completion of the COF formation in water (Fig. S17). It was observed that TpPa-1 formation was initiated after 6 h of heating the mixture containing 1,3,5-triformylphloroglucinol and *p*-phenylenediamine. After 18 h, the PXRD pattern distinctly showed the presence of the first peak corresponding to the 100 plane of TpPa-1 with the disappearance of the reactant peaks in the mixture, thereby confirming the completion of the reaction. The acetic acid used for the COF synthesis essentially lowers the pH of the medium thereby increasing the solubility of the diamines and eventually enhancing the reaction rate. Thus, the overall retention in the crystallinity, surface area and porosity of the hydrothermally derived COFs may be attributed to the improved solubility of the precursor amines in the reaction medium and also to the hydrogen-bonding environment provided by the acetic acid–water medium, which is well known to favour reversible dynamic covalent bonding, a factor very necessary for the COF formation.

## 4. Conclusions   

In summary, the present study focuses on water-mediated synthesis of COFs based on Dynamic Covalent Chemistry, which includes imine bond formation in water. We report the synthesis of six water-based, highly crystalline COFs, which ascertains the versatility of this synthetic technique. The overall retention in the crystallinity, surface area and porosity of the hydrothermally derived COFs may be attributed to the facile access to the Schiff base reaction in water, which favours the reversible dynamic covalent bonding necessary for the COF formation. Thus, this work reintroduces the command of the dynamic covalent principles for large-scale synthesis of COFs in water and eliminates the necessity of using organic solvents, otherwise used for COF synthesis.

## Supplementary Material

Crystal structure: contains datablock(s) COF-Benzidine_AA_vasp-PBE. DOI: 10.1107/S2052252516013762/yc5008sup1.cif

Supporting figures and tables. DOI: 10.1107/S2052252516013762/yc5008sup2.pdf
