# Supplementary material for: Constructing covalent organic frameworks in water via dynamic covalent bonding
Source: IUCrJ. 2016 Sep 14;3(Pt 6):402–7. doi: 10.1107/S2052252516013762 (PMC5094442; doi:10.1107/S2052252516013762)
Supplement: Supplementary file 2 [file m-03-00402-sup2.pdf]

IUCrJ

Volume 3 (2016)

Supporting information for article:

Constructing covalent organic frameworks in water *via* dynamic covalent bonding

Jayshri Thote, Harshitha Barike Aiyappa, Raya Rahul Kumar, Sharath Kandambeth, Bishnu P. Biswal, Digambar Balaji Shinde, Neha Chaki Roy and Rahul Banerjee

Section	Name	Page No
S1	General Information	2
S2	Synthetic Procedures	3
S3	Structural Modeling and Atomic Coordinates of COFs	3
S4	Powder X-Ray Diffraction Analysis (PXRD)	6
S5	FT-IR Spectra	8
S6	^{13}C CP/MAS NMR Spectra	9
S7	Stability test for water synthesized COFs	10
S8	Thermo Gravimetric Analysis (TGA)	12
S9	Transmission Electron Micrograph (TEM)	13
S10	Pore Size Distribution	13
S11	H_2 Gas Adsorption Studies	14
S12	CO_2 Gas Adsorption Studies	14
S13	Water Adsorption Studies	15
S14	PXRD patterns of reaction progress of TpPa-1	15

S1. General Information

The reactant 1,3,5-triformylphloroglucinol (Tp) was synthesized using previously reported procedure (Chong, J. H. et al., *Org. Lett.* **2003**, *5*, 3823). All the other reagents and solvents were commercially available and used as received. Powder X-ray diffraction (PXRD) was recorded by means of Phillips PANalytical diffractometer using Cu K_α radiation (λ = 1.5406 Å) instrument at a scan speed of 1° min^{-1}. The Fourier transform infrared (FT-IR) spectra were recorded in the 600-4000 cm^{-1} region using a Diamond ATR (Golden Gate) equipped Bruker Optics ALPHA-E spectrometer. The Thermogravimetric analyses (TGA) were carried out under N_2 atmosphere using SDT Q600 TG-DTA analyzer at a heating rate of 10 ºC min^{-1} and temperature range of 30-

900 °C. The SEM images were obtained using Zeiss DSM 950 scanning electron microscope and FEI, QUANTA 200 3D Scanning Electron Microscope equipped with tungsten filament as electron source operated at 10 kV. FEI Tecnai G2 F20 X-TWIN TEM at an accelerating voltage of 200 kV was used for recording TEM images. The TEM samples were prepared by dropcasting the sample (dispersed in from isopropanol) onto the copper grids TEM Window (TED PELLA, INC. 200 mesh).All the gas adsorption experiments (up to 1 bar) were performed on a *Quantachrome Autosorb-iQ2* automatic volumetric instrument at 298 K, 77 K and 273 K for water, N_2 and CO_2 respectively. Solid state NMR (SSNMR) was taken in a Bruker 300 MHz NMR spectrometer and Ligand NMR data were taken in Bruker 200 MHz NMR spectrometer. The recording was carried at different positions in order to verify the consistency of the measurement.

S2. Synthetic Procedures

General method for the synthesis of TpPa-1, TpPa-2, TpBD, TpFn, DAAQ and TpBPy in water

3 mmol of 1,3,5 triformylphloroglucinol (Tp) (0.63 g) and 4.5 mmol equivalent corresponding amine: *p*-phenylenediamine (Pa-1) (0.48 g,) / 2, 5-dimethyl-*p*-phenylenediamine (Pa-2) (0.61 g) / biphenyl-4, 4’-diamine (BD) (0.83 g) / 2, 7- diaminofluorene (Fn) (0.58 g) / 2, 6-diaminoanthraquinone (AQ) (1.02 g) / 2, 2’-bipyridine 4, 4’-diamine (Bpy) (0.837 g) are taken in water-acetic acid medium (36 ml: 18 ml). The mixture is sonicated for 15 min to achieve a homogenous suspension. The mixture is heated at 120 °C for 3 days using teflon lined autoclave using a programmed oven. After this time interval, the COF powders were filtered out, washed with water, followed by ethanol for 2-3 times and finally dried under vacuum at 150 °C for 12 hours (Isolated yield ~70-80%).

S3. Structure Modeling and Atomic Coordinates of TpFn

Details of Materials Studio (ver.6.0): Materials Studio (ver.6.0) suite of programs by Accelrys was used for the molecular modeling of the COF. A hexagonal unit cell with space group P6/mmm was initially used for the structural modeling. In order to calculate the exact space group and unit cell values Pawley refinement was done, using the MS Reflex Plus module. The refinement was applied to the calculated lattice, producing the refined PXRD profile.

Table S1 Fractional atomic coordinates for the unit cell of TpFn

Atom	x	y	Z
O1	O	1.29831	0.56181
N1	N	1.191	0.60951
C1	C	1.31497	0.61103
C2	C	1.22626	0.59364
C3	C	1.27883	0.63014
O2	O	1.43819	0.7365
N2	N	1.39049	0.58149
C4	C	1.38897	0.70394
C5	C	1.40636	0.63262
C6	C	1.36986	0.64869
O3	O	1.2635	0.70169
N3	N	1.41851	0.809
C7	C	1.29606	0.68503
C8	C	1.36738	0.77374
C9	C	1.35131	0.72117
O4	O	0.70169	0.43819
N4	N	0.80906	0.39451
C10	C	0.68503	0.38897
C11	C	0.77374	0.40636
C12	C	0.72117	0.36986
O5	O	0.56181	0.2635
N5	N	0.60951	0.41851
C13	C	0.61103	0.29606
C14	C	0.59364	0.36738
C15	C	0.63014	0.35131
O6	O	0.7365	0.29831
N6	N	0.58523	0.19259
C16	C	0.70394	0.31497
C17	C	0.63262	0.22626
C18	C	0.64869	0.27883

C19	C	0.58535	0.44649
C20	C	0.61853	0.50111
C21	C	0.59845	0.53514
C22	C	0.54441	0.5139
C23	C	0.51161	0.53953
C24	C	0.52412	0.59109
C25	C	0.48439	0.6036
C26	C	0.43121	0.5643
C27	C	0.41847	0.51172
C28	C	0.45817	0.49981
C29	C	0.45544	0.4473
C30	C	0.51152	0.45965
C31	C	0.53064	0.42538
C32	C	0.86297	0.42235
C33	C	0.88353	0.3884
C34	C	0.93749	0.4076
C35	C	0.97105	0.46152
C36	C	0.95057	0.49516
C37	C	0.89733	0.47692
C38	C	0.55968	0.14237
C39	C	0.50503	0.12022
C40	C	0.47167	0.06601
C41	C	0.49364	0.03384
C42	C	0.56116	0.01279
C43	C	0.54794	0.05592
C44	C	0.58155	0.10942
C45	C	1.03284	0.50155
C46	C	1.07218	0.48944
C47	C	1.1243	0.52945
C48	C	1.13775	0.58252
C49	C	1.0976	0.59486
C50	C	1.04612	0.55486
C51	C	0.99609	0.55712
C52	C	1.46536	0.96463
C53	C	1.41377	0.92564
C54	C	1.40116	0.87339
C55	C	1.44042	0.85945
C56	C	1.49305	0.89923
C57	C	1.50503	0.95087
N7	N	0.39049	0.58149
C58	C	1.43121	0.5643

C59	C	1.49364	1.03384
C60	C	1.56116	1.01279
C61	C	0.46536	-0.03537
C62	C	0.50503	-0.04913

S4. Powder X-Ray Diffraction Analysis (PXRD)

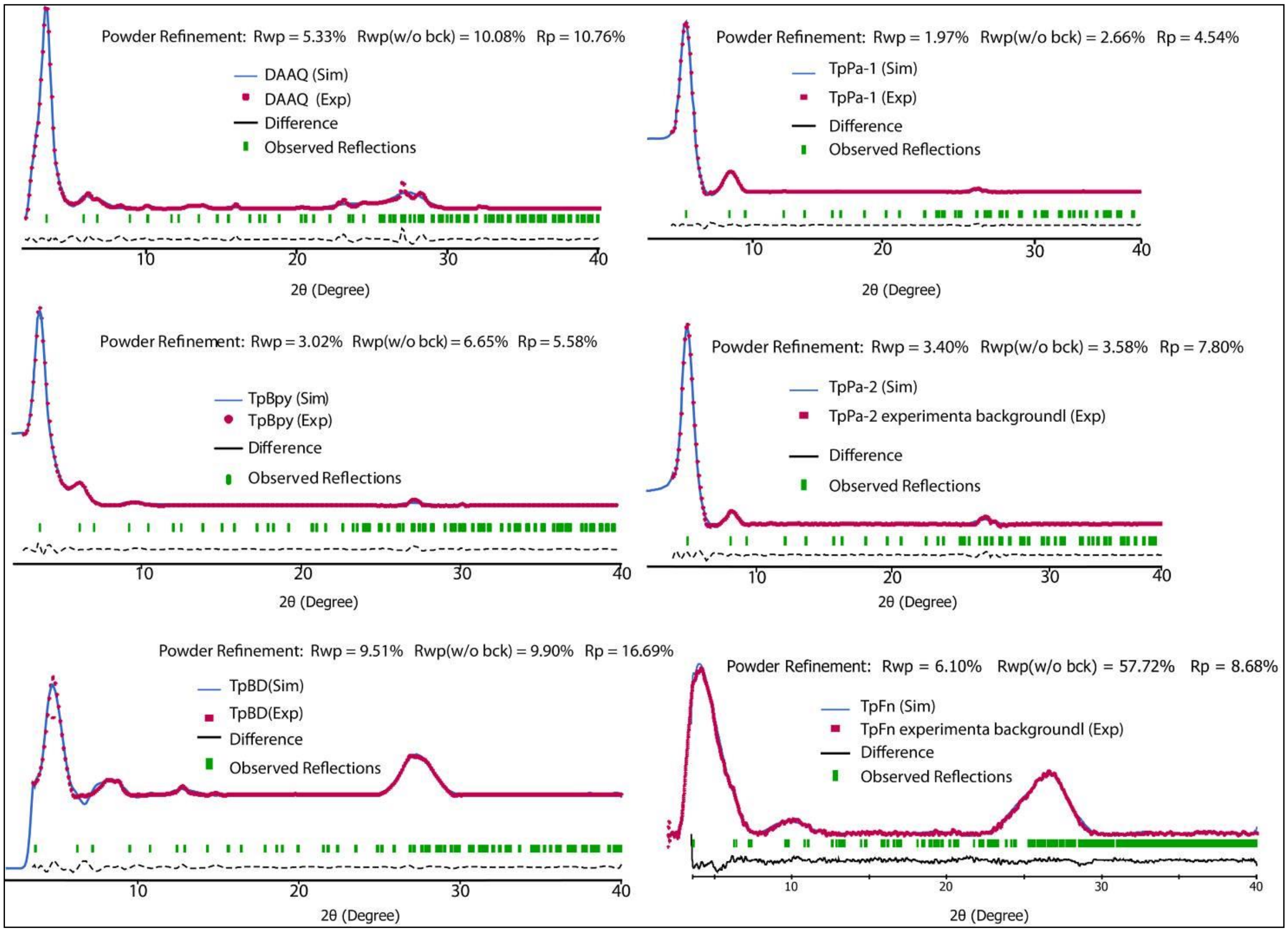

Figure S1 Comparative PXRD patterns of the as-synthesized COFs in water and their Pawley refinements.

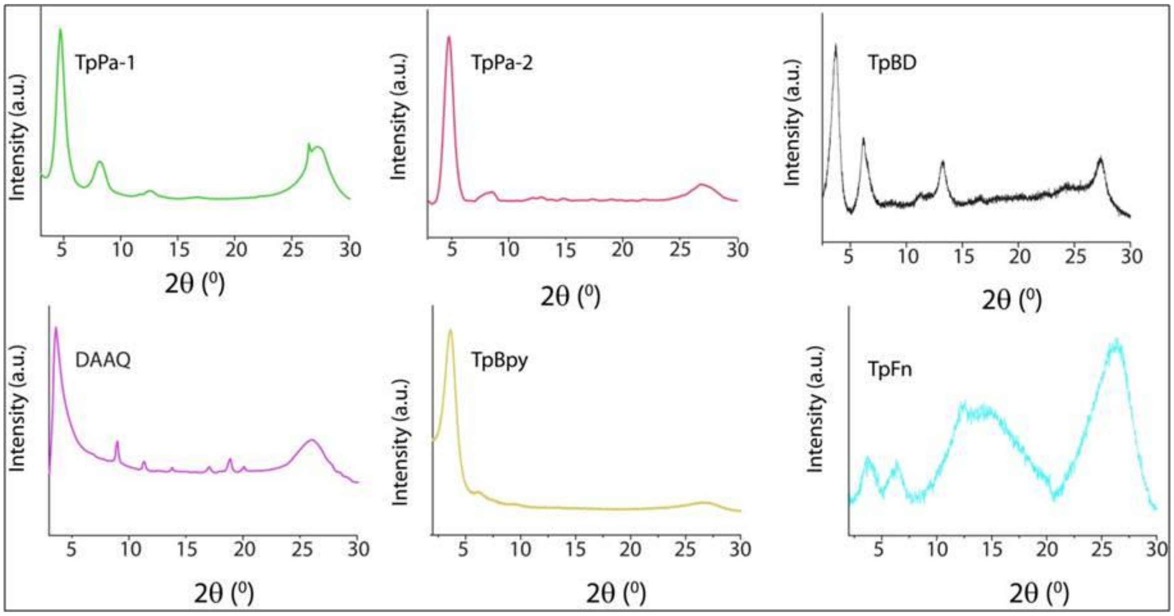

Figure S2 PXRD patterns of the COFs synthesized *via* conventional schlenk seal-tube technique using mesitylene-dioxane solvent system.

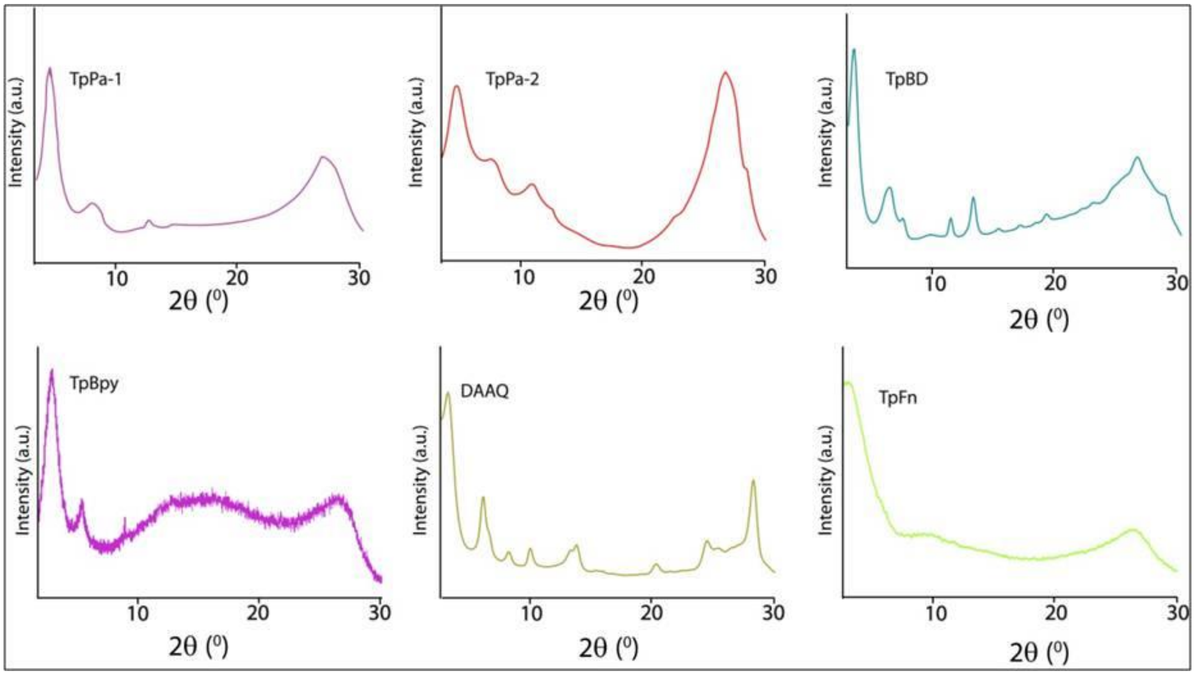

Figure S3 PXRD spectra of the COFs synthesized via mechanochemically grinding technique.

S5. FT-IR Spectra

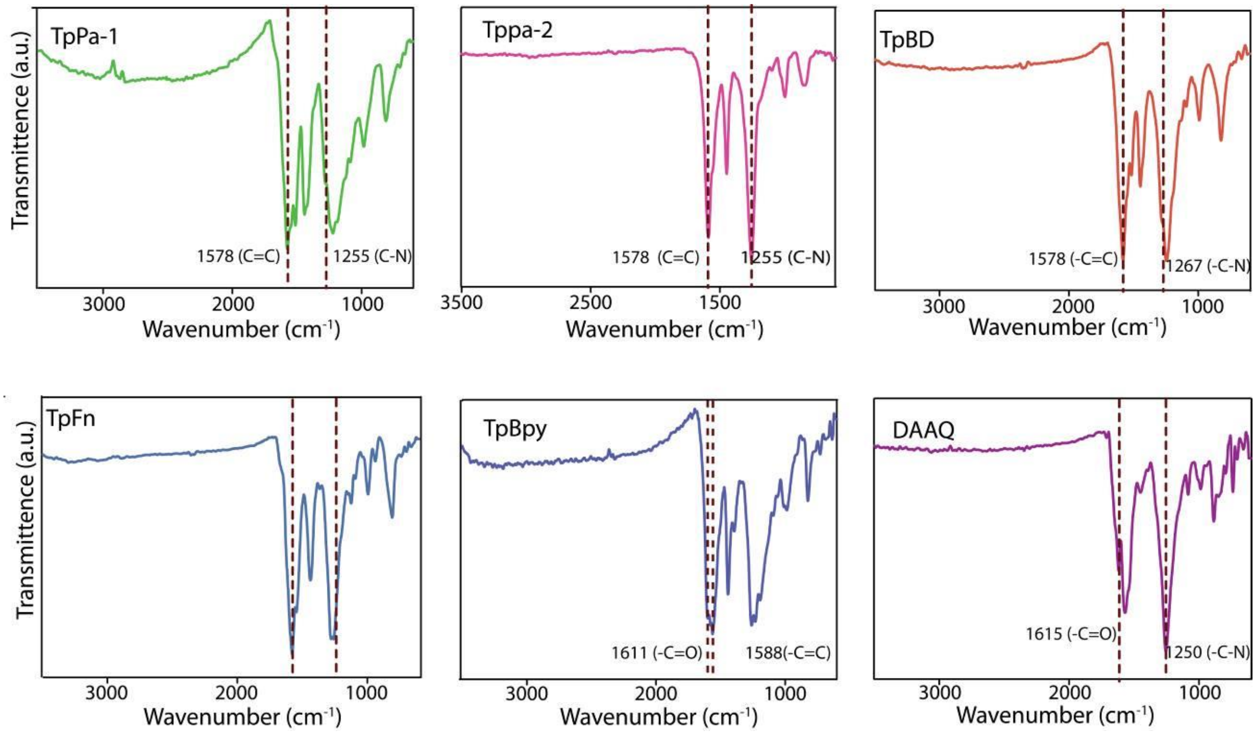

Figure S4 Comparative FT-IR spectra of COFs synthesized in water.

S6. ^{13}C CP/MAS NMR Spectra

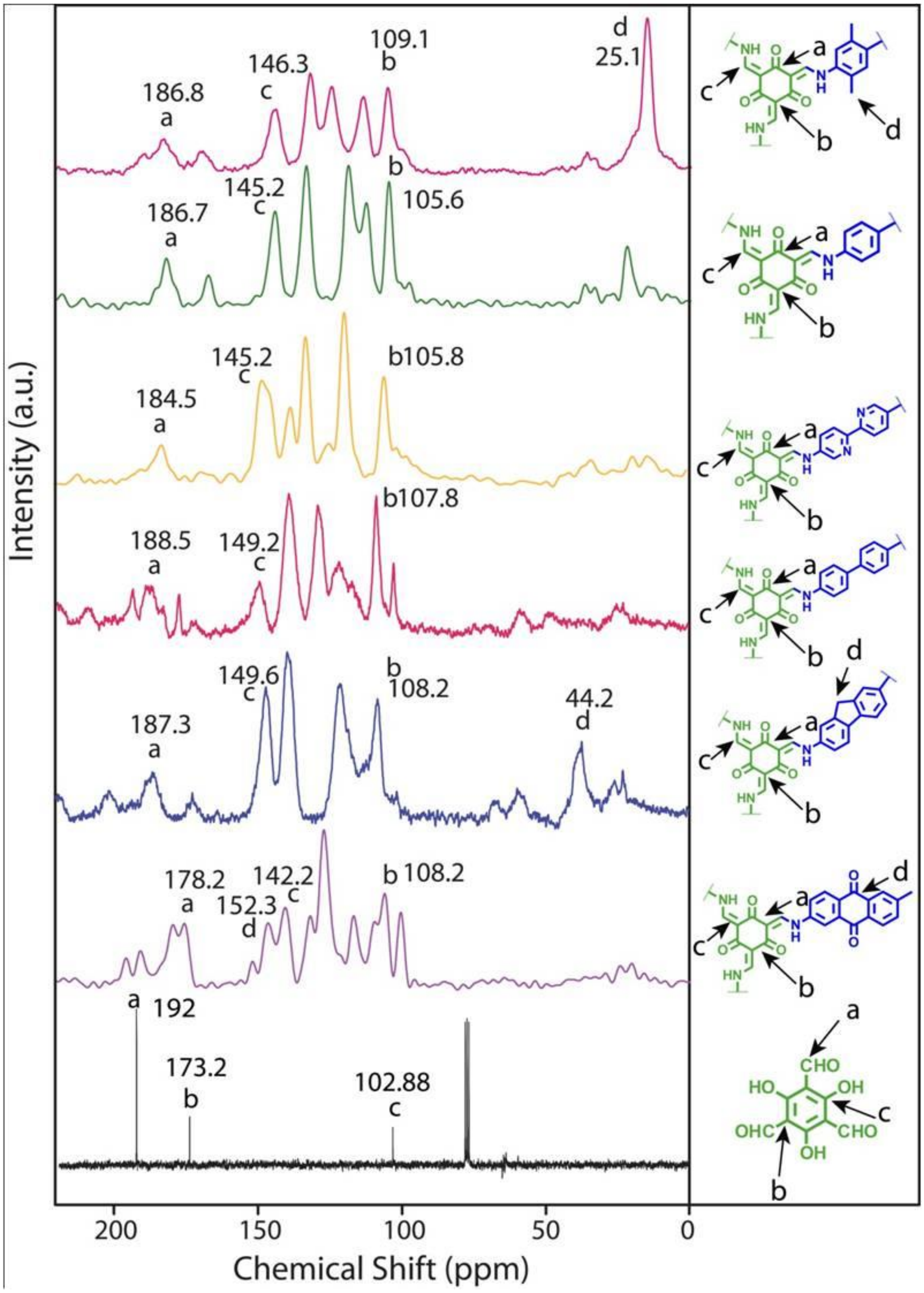

Figure S5 ^{13}C CP/MAS spectra of Tp and COFs synthesized in water.

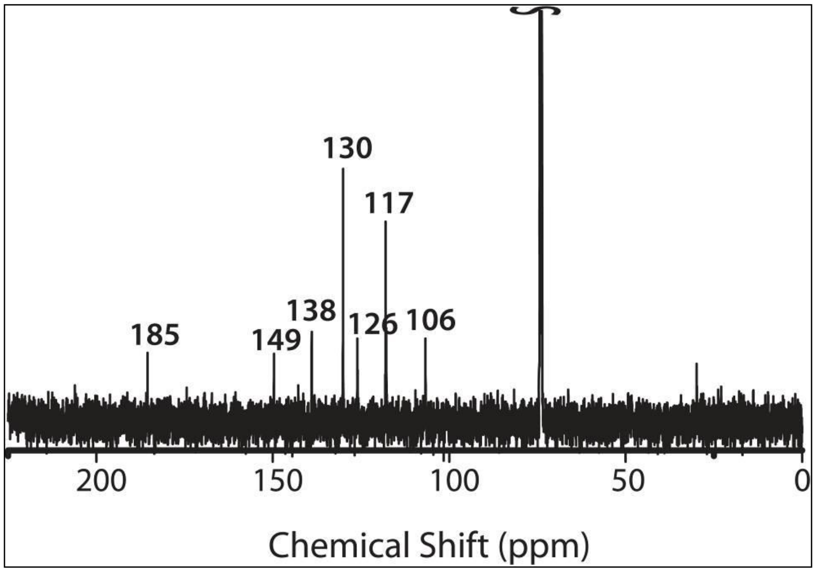

Figure S6 ^{13}C NMR spectrum of monomer 2 in 1,1,2,2-tetrachloroethane-d_2**.**

S7. Stability test for water synthesized COFs

Experimental details

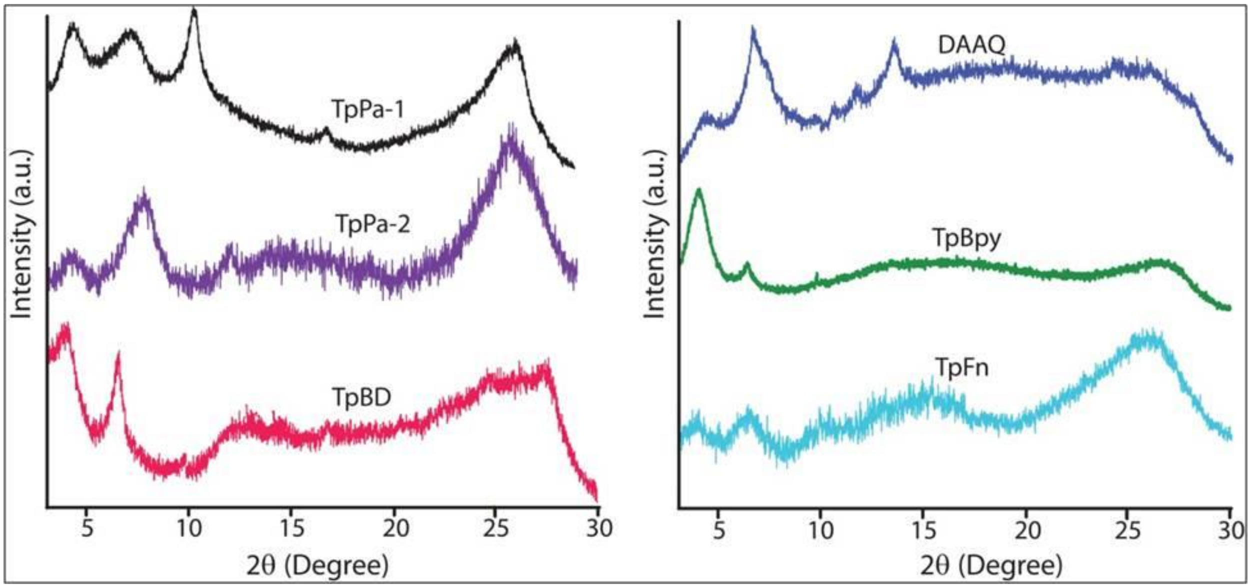

Figure S7 PXRD patterns of water synthesized COFs after treatment with 9N HCl for 3 days.

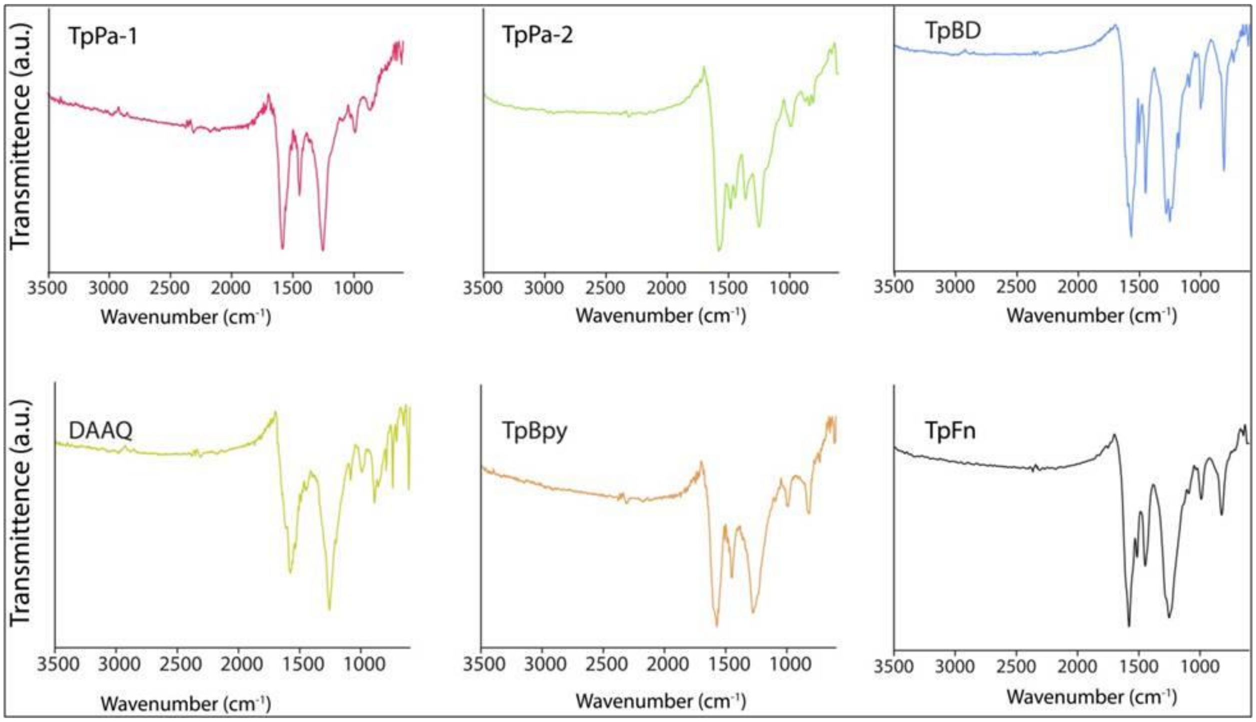

Figure S8 FT-IR spectra of water synthesized COFs after treatment with 9N HCl for 3 days.

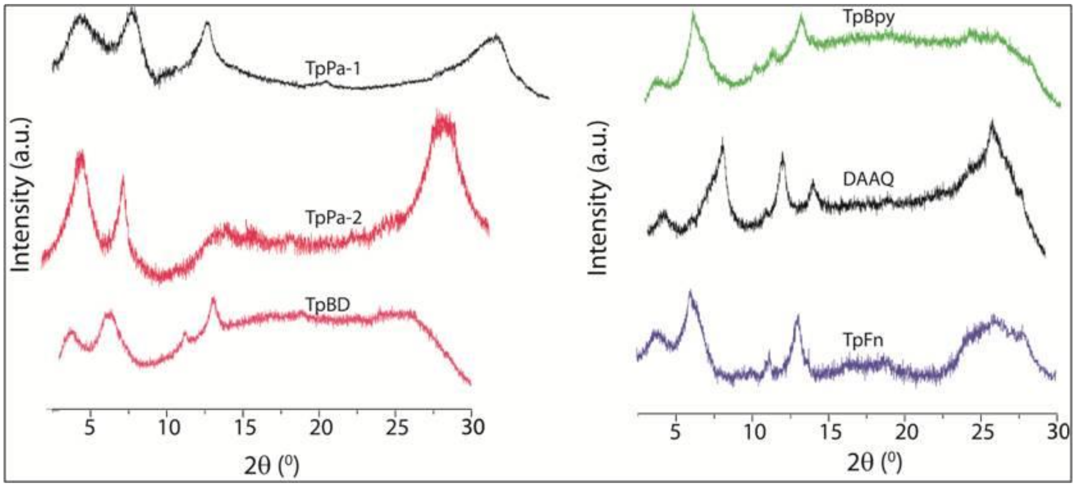

Figure S9 PXRD patterns of water synthesized COFs after treatment with 3N NaOH for 3 days.

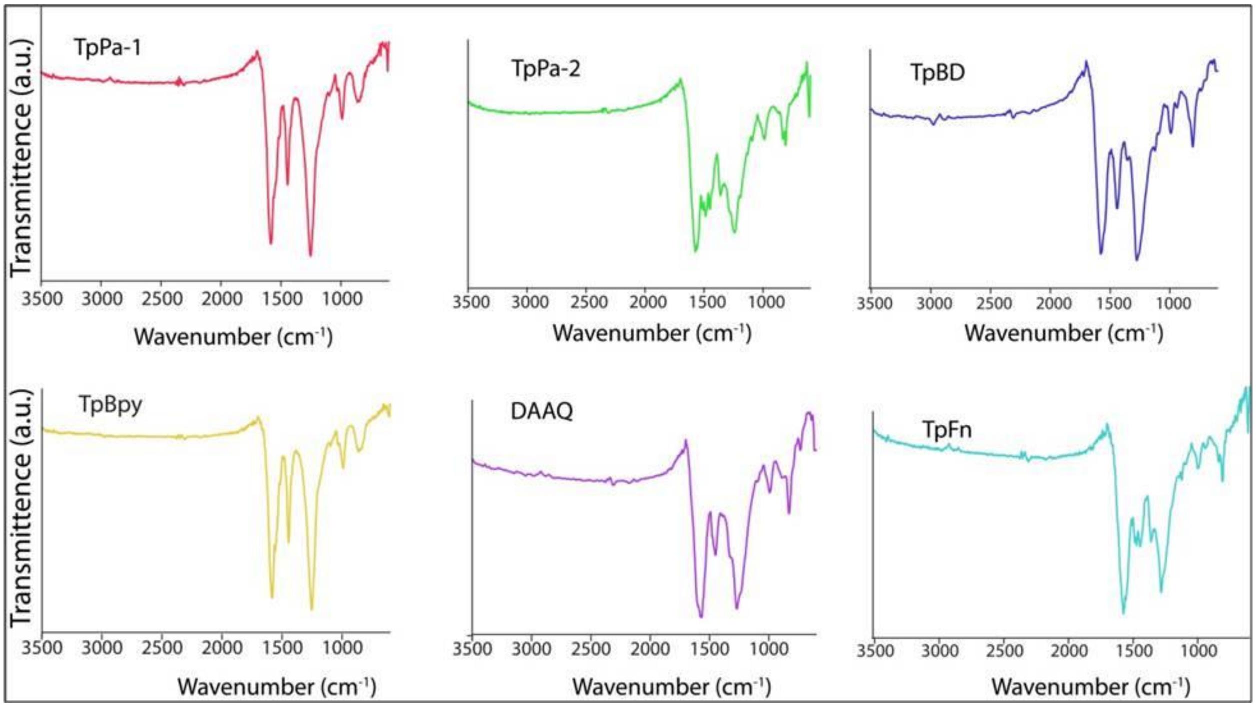

Figure S10 FT-IR spectra of water synthesized COFs after treatment with 3N NaOH for 3 days.

S8. Thermo Gravimetric Analysis (TGA)

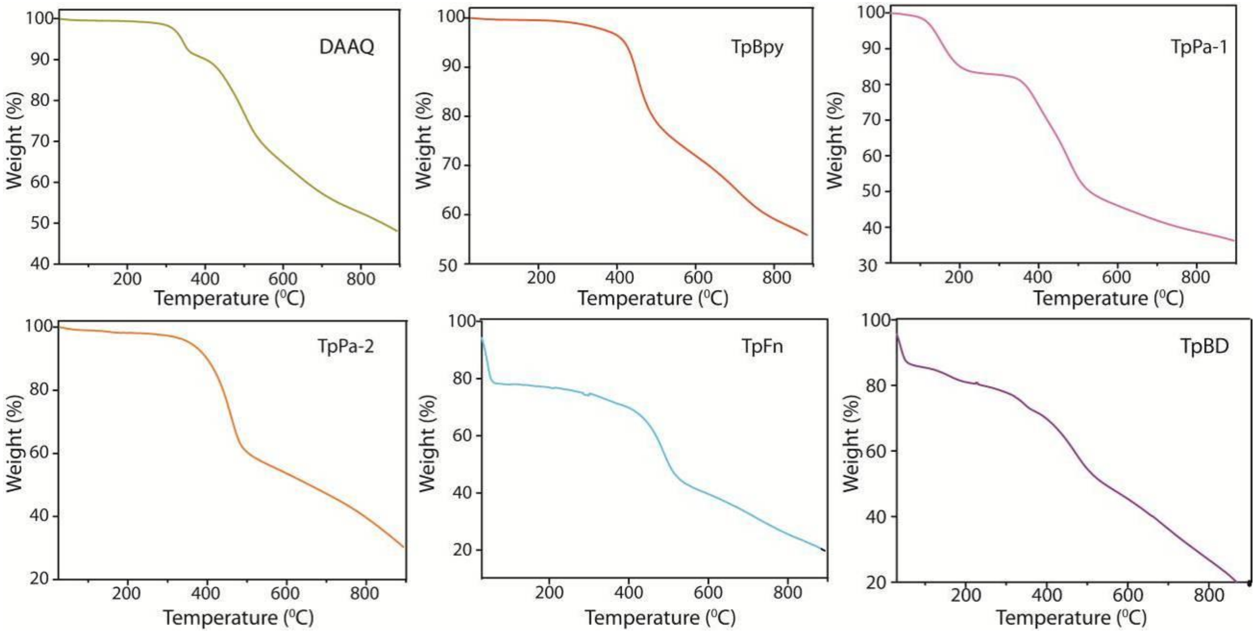

Figure S11 TGA profiles collected under N_2 atmosphere of COFs synthesized in water.

S9. Transmission Electron Micrographs (TEM)

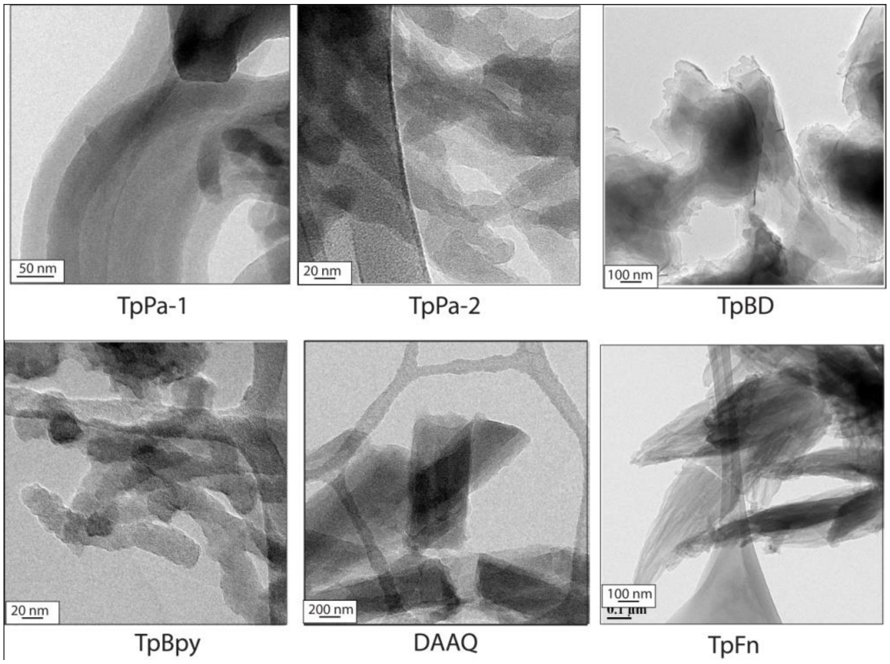

Figure S12 TEM images of COFs synthesized in water.

S10. Pore Size Distribution

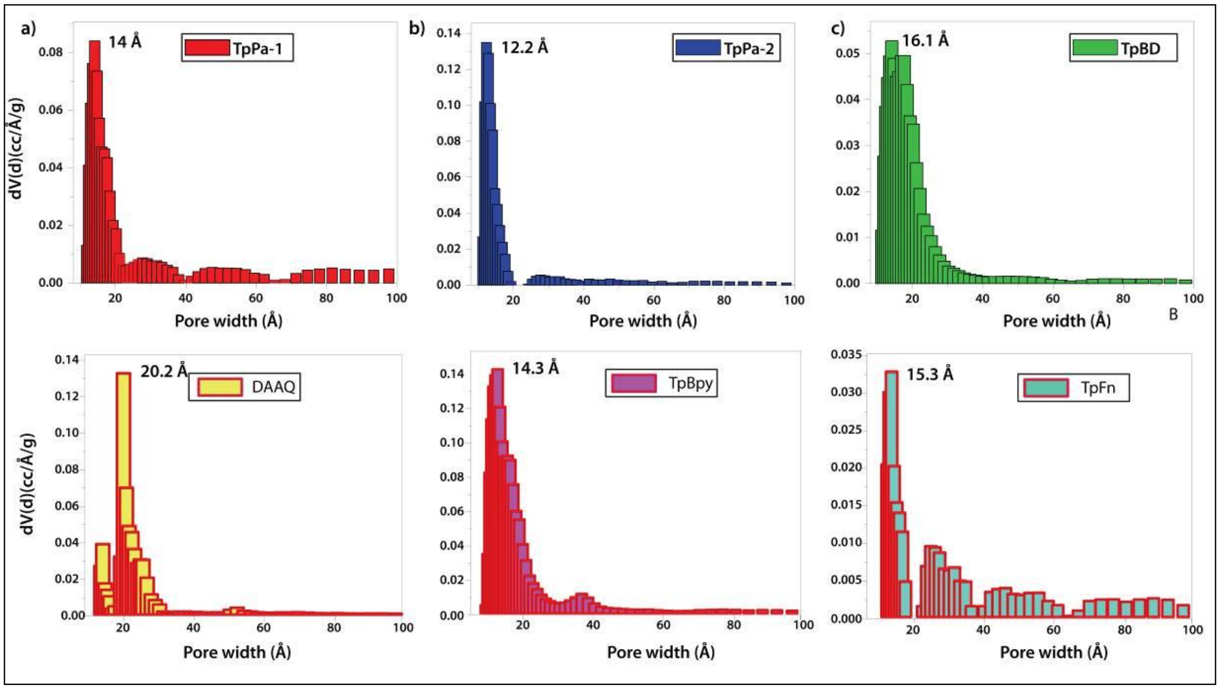

Figure S13 Pore size distribution of COFs synthesized in water.

S11. H_2 Adsorption Studies

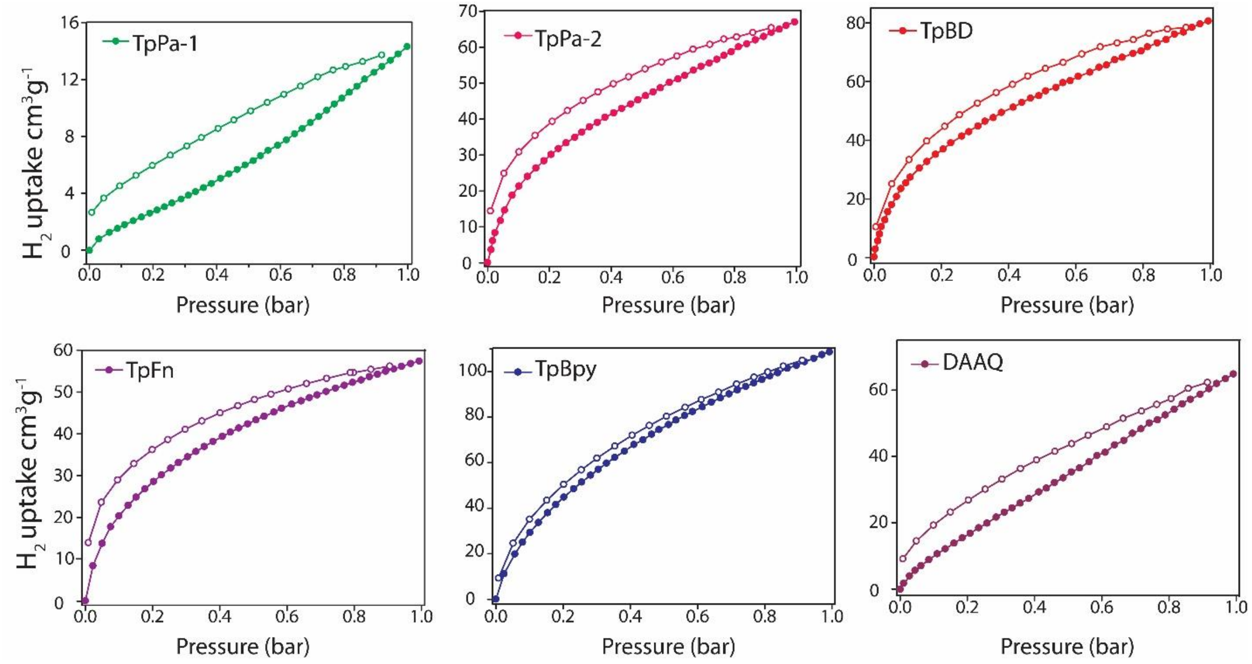

Figure S14 H_2 adsorption profiles of COFs synthesized in water.

S12. CO_2 Adsorption Studies

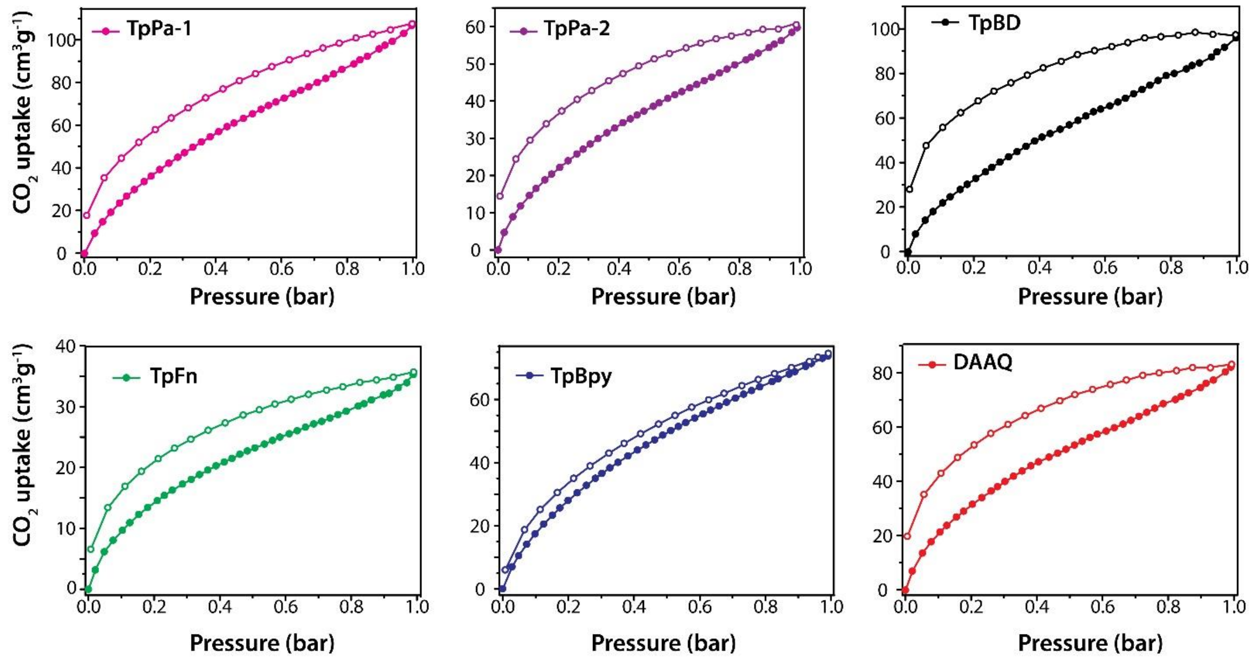

Figure S15 CO_2 adsorption profiles of COFs synthesized in water.

S13. Water Adsorption Studies

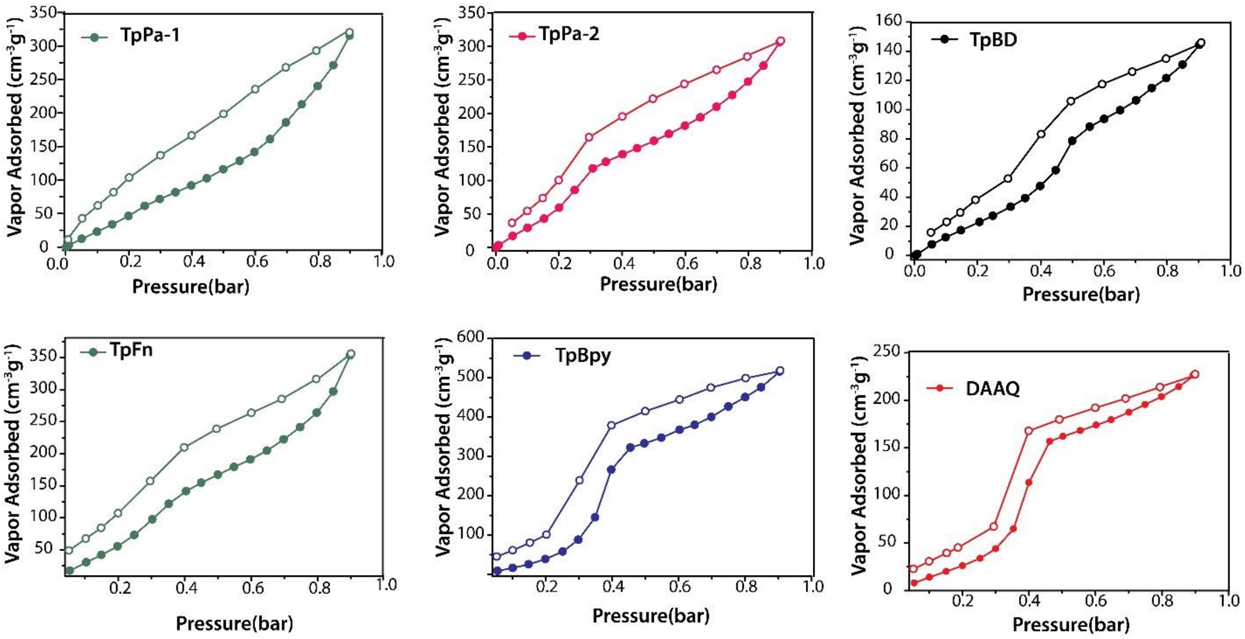

Figure S16 Water adsorption profiles of COFs synthesized in water.

S14. Time dependent PXRD patterns for monitoring the reaction progress of TpPa-1 in acetic acid-water medium

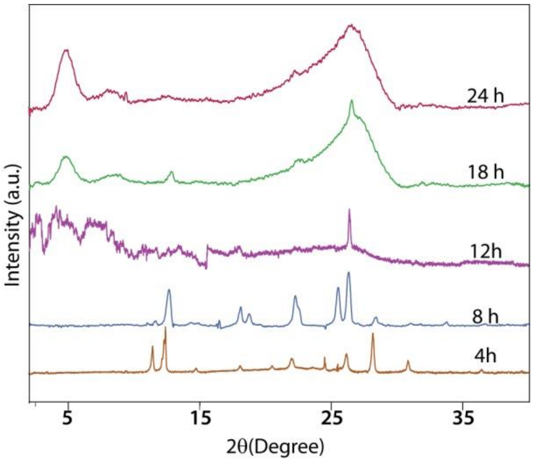

Figure S17 PXRD patterns of reaction progress of TpPa-1 in water– acetic acid medium.
